# What is the accuracy of the surgical guide in the planning of orthognathic surgeries? A systematic review

**DOI:** 10.4317/medoral.25042

**Published:** 2021-09-25

**Authors:** Maria Eduarda Pereira Goulart, Thaís Cargnino Biegelmeyer, Larissa Moreira-Souza, Celso Ricardo Adami, Fernando Deon, Isadora Luana Flores, Thiago Oliveira Gamba

**Affiliations:** 1 DDS. School of Dentistry, University of Caxias do Sul, Caxias do Sul, Brazil; 2 DDS, MSc. School of Dentistry, University of Campinas, Piracicaba, São Paulo, Brazil; 3 DDS, MSc. School of Dentistry, University of Caxias do Sul, Caxias do Sul, Brazil; 4 DDS, PhD. School of Dentistry, Federal University of Rio Grande do Sul, RS Porto Alegre, Brazil; 5 DDS, PhD. School of Dentistry, University of Caxias do Sul, Caxias do Sul, Brazil

## Abstract

**Background:**

To investigate the true accuracy of the surgical guide in the planning of orthognathic surgeries, which are performed worldwide.

**Material and Methods:**

A systematic search was conducted in the PubMed database, Web of science, Scopus and Embase, covering August 2020 to January 2021. Studies that included patients with dentofacial deformity including anteroposterior, vertical and asymmetry problems who were undergoing an orthognathic surgery procedure were included; QUADAS-2 was used to determine the risk of bias by analyzing the quality of the studies. A PRISMA (flowchart) was created to show the study selection, keywords, nomination processes, and inclusion and exclusion criteria.

**Results:**

Eleven studies were selected for qualitative and quantitative synthesis. All studies evaluated described high precision of the surgical guide, where the lowest error values were represented by the CAD/CAM technique.

**Conclusions:**

The planning and printing errors related to the guide were all less than 2 mm, and the absolute averages of the errors related to virtual planning in the analysis of the different plans were less than 1 mm. Finally, the measurement of the ANB angle obtained equivalent results between the virtual planning and the traditional.

** Key words:**Orthognathic surgery, splints, data accuracy, 3-D printing.

## Introduction

Diagnostic accuracy consists in discriminating a disease from a healthy condition or defining different stages of a disease. Accordingly, accuracy can be used to assess the result of an orthognathic surgery (OS) in relation to its planning performed virtually using software and printing a surgical guide. Both tools can reproduce the virtual planning for correction of facial bone deformities (1).

Facial skeletal deformities and malocclusions have been corrected by OS. The inadequate relationship between maxilla and mandible can be the result of excess and/or deficiency in either maxilla or mandible development or even in both jaws. Facial skeletal deformities may result in dental occlusion disorders and phonation, breathing and articular and aesthetic problems (2). However, OS has functional and aesthetic goals of achieving class I dental occlusion and facial symmetry and proportion (3).

This surgical procedure can be simulated by the conventional method. Physical examination is first performed, where asymmetries, facial proportions and facial profile type are evaluated. The patient is classified as a straight, concave or convex profile type (4). Imaging examinations such as panoramic and cephalometric radiography are also required, in which prediction tracings are made. Once the OS involves the jaws, it is also necessary to use plaster dental models, which are attached to a dental articulator. The surgical simulation is completed by moving the bone tracings to the desired position (4).

However, with this conventional method, it is not possible to simulate surgery in 3-dimensions. Moreover, plaster dental models do not show the surrounding bony structures. Therefore, there is a limitation to visualizing the skeletal changes that occur during model surgery, which is essential in the treatment of complex craniomaxillofacial deformities (4). Other dimensional errors can occur during the steps of this conventional method, which can occur while obtaining the plaster models and/or during the mounting of the models in the articulator. In addition, errors may be observed on physical examination or on cephalometric analysis, which may also result in technique failures (5).

On the other hand, virtual planning in OS has overcome the limitations of the conventional method. Computed tomography (CT) images in a DICOM file (Digital Imaging and Communication in Medicine) have been used in addition to jaw scanning, which can be done by an intraoral scanner or by the plaster cast models obtained from a prosthetic laboratory (6). Moreover, imaging exams such as CT scan, which offers high-resolution three-dimensional images, permits a detailed visualization of bone structures and adjacent tissues, providing a better diagnosis and surgical planning and allowing patients to preview the final treatment outcome (7).

However, CT images have limitation such as imaging artefacts, since brackets and metal restorations may interfere with the visualization of important anatomical landmarks (8). On the other hand, the virtual method does not require many laboratory steps, as with the conventional one, and there is no need for the use of a facial arch, since it uses three-dimensional images, which allow the visualization of adjacent structures and their influence on occlusion. Also, the virtual surgery simulation uses three-dimensional images, which can simulate osteotomies, jaw replacement, intercuspidal control, and postoperative results shown on a computer screen (6) and stored in the software (5).

Because of this, the virtual planning technique is expected to be more accurate than the conventional one (5) and the use of a surgical splint is expected to provide faster surgeries. Given the state of the art, this systematic literature review aimed to investigate the true accuracy of the surgical guide in the planning of OS, which are performed worldwide.

## Material and Methods

- Protocol and registration

The protocol of this systematic review was developed following the preferred reporting items for systematic reviews and meta-analyses (PRISMA) and the study was registered at international prospective register of systematic reviews (PROSPERO) — protocol: # CRD42020152755. This systematic review was reported according to the PRISMA checklist.

- Problem specification

Initially, the following review question was used to establish a search strategy: What is the accuracy of the surgical guide in the planning of orthognathic surgeries?

- Data source and search strategy

The literature search was conducted in the PubMed database (Medline), Web of Science, Scopus and Embase from August 2020 to January 2021. The EndNote Basic® software (Thompson Reuters, New York, NY) was used, and duplicated hits were removed. Appropriate truncation and word combinations were selected with the support of a health sciences librarian and were adapted for the database. The keywords applied in the research were: orthognathic surgery, guide (splint), and rapid prototype (3D printing, CAD/CAM and accuracy).

- Eligibility criteria

Inclusion criteria: Studies in which the primary aim was to evaluate the accuracy of the surgical guide for OS planning using assessments in CBCT in humans (in vivo and cadavers) were considered. Studies that had patients diagnosed with maxillofacial skeletal deformities as sample were selected. Only studies published in English were selected. No date and year filters were used to select the studies.

Exclusion criteria: Studies characterized as narrative reviews, systematic reviews, expert opinions, letters, case reports, book chapters, and conference abstracts were not considered. Studies regarding the use of surgical guides for other dental procedures such as dental implants and endodontic treatment were excluded. In vitro studies were excluded, as well as the studies that evaluated the presence of patients diagnosed with malignant lesions, trauma to facial bones, congenital malformations affecting facial development, patients diagnosed with obstructive sleep apnoea and temporomandibular joint disorder. Finally, studies that did not mention the use of the surgical guide as a tool for OS planning were not considered.

- Publication retrieval

A two-phase selection of the studies was conducted. In phase 1, two authors (MEG and TCB) independently reviewed the titles and abstracts of all references. In phase 2, full texts were independently reviewed by the same authors. They selected the studies that met the previously determined inclusion and exclusion criteria. Any disagreement was resolved by means of discussion. When mutual agreement between the reviewers was not reached, a third author (TOG) became involved to make a final decision. Studies selected should mention all the inclusion criteria described above.

Population: Patients were those with dentofacial deformity including anteroposterior, vertical and asymmetry problems who underwent an OS.

Study design: The selected studies were double-blind randomized controlled clinical trials, experimental, prospective observational, and prospective randomized trials, pilot studies, and retrospective observational studies.

Methods: Studies that assessed the accuracy of the surgical guide to be applied in OS in human patients or even in cadavers. All studies comparing values between pre and post OS planning.

Index test: Studies that presented the possibility of clinical application in patients undergoing OS and protocols can be reproduced in patients with indication for this surgery to correct skeletal alterations.

Reference standard: Accuracy values between the measures or angles obtained from the initial planning and the results of the OS were analyzed for each study.

Result: Statistical tests were assessed in each study to calculate the accuracy and/or the difference between the planning and final position, after OS, which were: Bland-Altman tests, Fisher exact test, Mann-Whitney test, unpaired Student t-test, Wilcoxon rank sum test, Wilcoxon test for paired samples, two-tailed test, parametric one-sample t-test, Whelch t-test, two one-sided test, paired t-test. Procrustes analysis and euclidean distance between two sets of coordinates were performed to calculate landmark error and lower model position wafer error, respectively.

Language: English

- Quality assessment and data synthesis

Regarding the quality assessment of the selected studies, three authors of the present study (MEG, TCB and TOG) used QUADAS 2 tool (Quality Assessment of Diagnostic Accuracy Studies-2) and assessed the risk of bias of the studies according to the following criteria: low, high or unclear, in four domains: (I) sample selection, (II) index test, (III) reference standard and (IV) flow and timing. The domains I, II, and III were re-assessed to investigate their applicability. For such analysis, a QUADAS 2 protocol (Table 1) was adapted according to the problem specification (9).

**Table 1:**
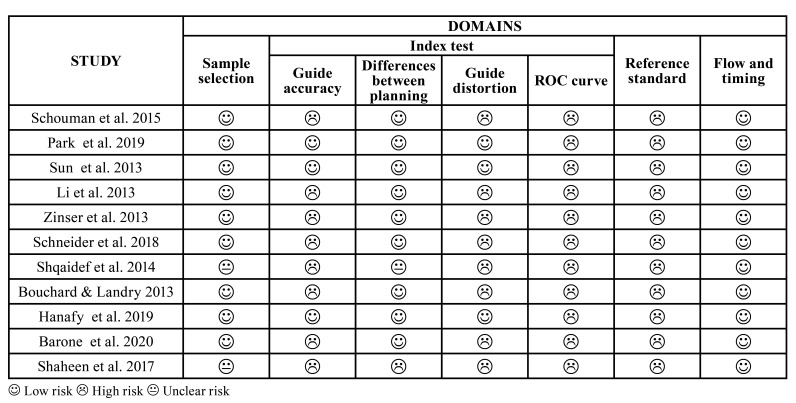
Risk of bias according to QUADAS-2 for domains: sample selection, index test, reference standard, flow and timing.

A final evaluation was made to assess the quality of the studies. Prognostic questions were created and adapted according to the guidelines of systematic reviews. Any disagreements in the analysis of the answers were determined with consensus among the three evaluators. The two reviewers (MEG, TCB) extracted and tabulated the characteristics of each study; subsequently, a third reviewer (TOG) confirmed the authenticity of the data.

- Risk of bias in individual studies

The analysis of the quality of the selected studies were evaluated by QUADAS 2. Two authors of the present study (MEG, TCB), independently of this tool, classified the selected studies according to the answers, yes, no or unclear. Possible disagreements were resolved by the third evaluator (TOG).

- Summary measures

The primary outcome of the selection of studies was to obtain the accuracy of the surgical guide in the planning of OS or the comparison between the final surgical outcome in relation to the initial planning. In addition, any measure aimed at comparing the accuracy of the initial planning with the final one was analyzed, and all its variables and values of the statistical tests shown in the studies were evaluated. In addition, possible distortion values of the surgical guides were investigated.

- Risk of bias across studies

Through the planning of CT in humans (in vivo or cadavers), the result of the OS compared to the initial planning was analyzed. The heterogeneity of the different methodologies of the different studies was compared with respect to their measurement evaluated, software used, statistics and risk of bias.

## Results

- Study selection

**Figure 1:**
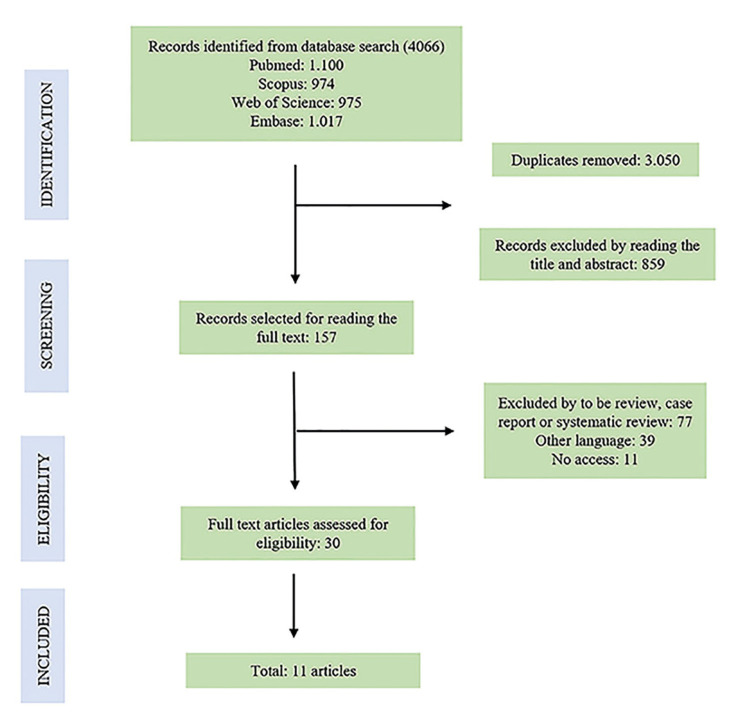
Flow diagram according to the PRISMA statement presenting the study selection process with the number of publications retrieved included and excluded for systematic review about the accuracy of the surgical guide.

After the references were initially identified by abstract and title, 157 references remained after phase 1 of the selection process. After phase 2 of the selection process, the final included sample consisted of 11 studies. Details about the selection process can be found in Fig. 1. Due to the disagreement of statistical tests for accuracy analysis, no meta-analysis was performed. Finally, 11 studies were included for qualitative and quantitative synthesis.

- Study characteristics

Eleven studies were selected, six in Europe (two in Germany, two in Belgium, one in Switzerland and one in Italy), one in North America (Canada), three in Asia (two in China and one in South Korea) and one in Africa (Egypt). The studies were published in English, between 2013 to 2020.

Regarding the types of studies, one experimental study, two retrospective observational, one randomized controlled clinical trial, four observational prospective, one randomized prospective and one pilot study were selected. Four studies evaluated the accuracy of splints or occlusal wafers generated by CAD/CAM for OS; one study developed and validated a new CAD/CAM model, while another compared versatility and accuracy of CAD/CAM products, analyzing surgical operations, intraoperative navigation and intermaxillary occlusal operations. In addition, four studies compared the accuracy of conventional and virtual surgical planning, through analysis of the wafers, surgical time and costs, including one that defined it by angles. Finally, one study printed surgical guides based on OS 3D planning and did a validation of accurate estimates in a large-scale comparative study of conventional analogous conFiguration in terms of absolute errors.

In contrast, in the analysis of the sample size, studies with 10 human cadaver heads and 20 (in two studies), 15, 6, 28, 21, 10, 23, 18 and 60 patients were observed. The analysis regarding surgical procedures in each study was: Le Fort I osteotomy, maxillary segmentation, bimaxillary surgery, Le Fort I high osteotomy, Le Fort I low – with an additional genioplasty; bimaxillary OS; Le Fort I; maxillary and mandibular combined osteotomy; maxillary advancement; impaction previously; impaction later; disimpaction previously; bilateral sagittal osteotomy surgery; bimaxillary surgery (and Le Fort I). A summary of the descriptive characteristics of the included studies is provided in Table 2

In addition, Table 3 describes for each included study the kind of computed tomography, planning software, scanner, type of guide, time of surgery to the follow accuracy results, which were:

1- Precision for maxillary planning transfer: <0.23 mm for CAD/CAM guides; <0.61 mm for waferless navigation; <1.1 mm for classic intermaxillary occlusal guides (8).

2- In 58.3% of cases, the conventional guides showed inaccuracy problems (27.7-84.4%), and in the planned virtual guides 0% showed this lack of precision (10).

3- The maximum error was 0.88 mm, and the average error was 0.4 mm (which is smaller than the clinically relevant error margin of 0.5 mm) (11).

4- The error related to the surgical guide was less than 2 mm, confirming an accepTable accuracy of the digital guides (12).

5- The average difference between planned and executed movement in any direction was 0.1 mm (13).

6- The mean absolute maxillary position error was less than 1 mm, providing clinically accepTable accuracy in maxillary repositioning, and the maximum error was well controlled at 1.7 mm. The absolute errors between the planned and actual maxillary positions were 0.7 mm axially, 0.6 mm horizontally and 0.8 mm vertically (14).

7- The error in prototyped guide was up to 1.73 mm, but if this resulted from the rapid prototyping process present in the virtual digital splint, this needs further investigation (15).

8- In the conventional group, the mean difference in maxillary position between virtual simulation models and postoperative results was 0.78 mm, while in the modified group (virtual planning), it was 0.77 mm (16).

9- The difference between the planned and the actual bony surgical movement at the edge of the upper central incisor was 0.50 - 0.22 mm in the sagittal direction, 0.57 – 0.35 mm in the vertical and 0.38 – 0.35 mm in the horizontal (17).

10- The CAD/CAM group showed mean deviations of 0.26 mm vertically, 0.17 mm anteroposteriorly, and 0.07 mm mediolaterally, while the classic wafer group showed mean deviations of 1.45 mm vertically, 1.31 mm anteroposteriorly and 0.71 mm mediolaterally (18).

11- The measurements planned and those obtained after surgery were equivalent in group D (digital planning) and in group T (traditional planning), the analysis showed equivalence only for one of the measurements considered: angle between point A: subspinale and nasion (line AN) and point B: supramentale and nasion (line BN) (ANB) (19).

- Additional information

Some factors are important to report and can be related to the accuracy of the different studies selected. The main factor is that accuracy indicates the proximity of a measurement value with the standard reference. However, the selected studies actually showed precision rather than accuracy. Moreover, the presence of metal artefacts from restorations or plates may alter the measurement variation during the recording and fusion process. Also, possible errors resulting from the movement of maxillary and mandible rotation in planning may affect determination of the true accuracy. Finally, a centric relationship pattern is essential to decrease the chance of error during the acquisition of cone-beam computed tomography (CBCT) images and in the planning of orthognathic surgeries.

- Risk of bias within studies

In the risk of bias analysis of the studies, no study m*et al*l the methodological quality criteria according to QUADAS-2 (Table 1). For each study, item 1 of the QUADAS criteria -2 (Domain 1- Sample selection) was classified as “no”, since all patients selected in the sample had indication for OS, except for two studies where sample selection was not clear.

**Table 2:**
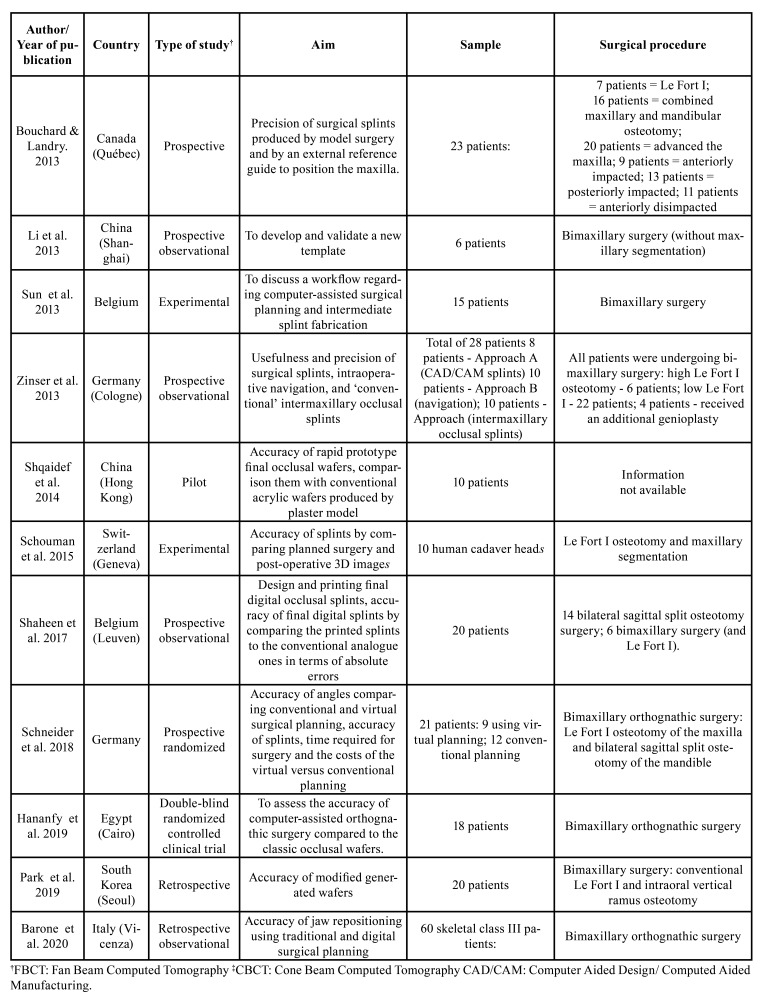
Descriptive general characteristics of the included studies.

**Table 3:**
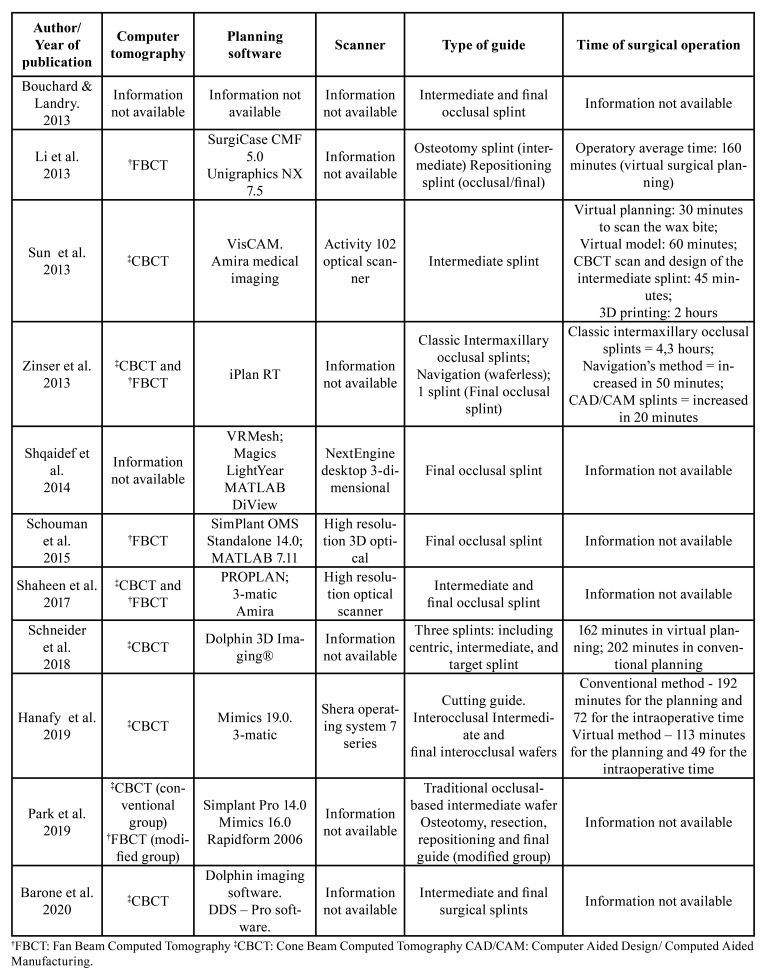
Characteristics of guide planning for orthognathic surgery of the included studies.

Regarding Domain 2 (Index Test), which requires the assessment of the accuracy found in each study, it was observed that only three of the studies found the accuracy and correlated possible guide distortion, but none of them used the most appropriate statistical test for accuracy analysis, which is ROC (receiver operator characteristic) curves.

In contrast, ten studies compared the difference between OS planning and the outcome, which represented a low risk of bias; only one of them was not clear and another did not evaluate this criterion. When assessed in relation to the reference standard, as a domain of risk of bias, a high risk of bias was evidenced in all studies, since the accuracy analysis was done in nine studies comparing the postoperative outcome to the surgical planning. This did not correspond to the best way to assess the accuracy result. It is recommended that ROC curves be used. Finally, in the analysis of the domain of flow and time bias risk, a low risk of bias was observed in all studies since there were appropriate intervals between the comparative patterns of planning prior to the result.

## Discussion

Various dental professionals around the world have been introducing rapid prototyping as a possible method for OS. This procedure recommends the use of virtual planning for the preparation of guides or splints that can help the oral surgeons to manage the correct position of the maxilla and/or mandible during the patient's surgery. This systematic review aimed to find through a selection of studies using exclusion and inclusion criteria studies that identify the true accuracy of surgical guides and virtual planning in relation to the final postoperative outcome (4,10).

After analysis of the PRISMA protocol, eleven studies from Europe, North America, Asia and Africa were selected. The European and North American countries commonly used virtual technologies and the digital systems were constantly upgraded. However, four of the selected studies were from Asia (China and South Korea) and Africa (Egypt), which are emerging regions with increasing funding in new technologies to compete with developed countries. Accordingly, it allows oral and maxillofacial surgeons to acquire a new option for OS planning (12,15).

The study sample size included 231 cases evaluated in total (Table 2). In the study published by Zinser *et al*. (8), 8 patients were submitted to virtual planning, 10 patients to the navigation technique and 8 patients to the conventional method using occlusal intermaxillary guides. As shown by Schneider *et al*. (10), virtual surgical planning was used in 9 patients, while the conventional method was used in 12 patients. Regarding the distribution between males and females, the study by Bouchard and Landry (13) included 19 female and 4 male patients. Studies with cadavers or even with only patient’s images were equivalent in relation to the sample size, and they had an average of 16.85 years. According to Barone *et al*. (19), 60 skeletal class III patients were selected, where 11 males and 19 females were submitted to traditional planning and 12 males and 18 females to digital planning.

The accuracy of the surgical planning compared with the postoperative results found in the present sample had errors of less than 2 mm, which are clinically accepTable. Schouman *et al*. (12) affirm that movements less than 2 mm are undetecTable to the naked eye or even cannot be identified by patients. The accuracy, which suggests the proximity of a measurement value to a standard reference, was not assessed by the selected studies, but rather precision was evaluated. We believe that this was because the authors did not use the correct statistical test, such as ROC curves. It can be determined whether the word “accuracy” can be changed to the word “precision” or if a different methodology can be used, such as the ROC curve statistical test to analyze the proposed results (12).

Five of the eleven selected studies assessed the surgical time required to perform guided surgery with different types of planning. According to Zinser *et al*. (8), OS performed by the virtual method had a longer duration compared to the conventional one, which lasted an average of 4.3 hours, increasing by 20 minutes using CAD/CAM guides and 50 minutes using the navigation method. On the other side, Schneider *et al*. (10) reported a 31% decrease in time in virtual planning (162 minutes) compared to conventional planning (202 minutes). Li *et al*. (14) highlighted an average operating time of 160 minutes when planning surgery was done virtually. According to Sun *et al*. (17) the CAD/CAM surgical planning method took 30 minutes to scan the wax bite, 60 minutes to take the virtual model, 45 minutes to CBCT scan and design of the intermediate splint and 2 hours to print it. Hanafy *et al*. (18) observed a time of 192 minutes for the conventional planning method and 72 minutes for the intraoperative time, while the virtual planning method (CAD/CAM) took 113 minutes for the planning and 49 for the intraoperative time. Independently of the time of surgical planning, it is important to emphasize that the use of guided surgeries can offer less risk of intraoperative complications and consequently better postoperative outcomes. In addition, the more experience the professional has with virtual planning and 3D software, the more planning time will decrease (14).

Knowledge in three-dimensional imaging examinations, such as CT, is important to achieve better performances in OS planning. Schouman *et al*. (12) and Li *et al*. (14) mentioned the use of fan-beam computed tomography (FBCT) in their studies. Zinser *et al*. (8), Schneider *et al*. (10), Shaheen *et al*. (11), Park *et al*. (16), Sun *et al*. (17), Hanafy *et al*. (18) and Barone *et al*. (19) reported CBCT as the imaging examination of choice. The other authors did not report the kind of CT used in their studies. In the present systematic review, we suggest the use of CBCT for the OS planning because the radiation dose used to acquire the volumes are lower than the radiation dose required by FBCT, which reduces the chance of a stochastic effect in patients (8,10,11).

The purpose of the surgical guides is to help the professional to achieve the previewed position of the jaws planned before the OS. Schouman *et al*. (12) and Shqaidef *et al*. (13) used only a final occlusal splint to perform the procedures, while Sun *et al*. (17) employed just an intermediate occlusal splint in their study. Park *et al*. (16), Li *et al*. (14), Zinzer *et al*. (8) Bouchard and Landry (13), Shaheen *et al*. (11), Hanafy *et al*. (18) and Barone *et al*. (19) combined both kinds of guides (intermediate and final). According to Zinser *et al*. (8), three surgical guides were applied using the CAD/CAM system, including a final occlusal guide, condyle establishment (centric guide) and a reference point of the skull. In addition, a surgical guide was not used in navigation surgery, and in the conventional method, only the final occlusal guide was used (8). Schneider *et al*. (10) described the use of centric, intermediate and final occlusal guide. We observed a wide range of techniques for virtual OS planning. However, independently of the technique, we believe the centralization of the median sagittal plane of the patient is essential, mainly when patients present with facial asymmetries. It can help to standardize the results and obtain the accuracy between what was planned and what achieved after the OS (10).

The risk of bias based on QUADAS-2 (Table 1) investigated the methodological quality of the studies selected for this systematic review.

According to the previous criteria of sample selection, none of the studies matched all domains. Only two studies were not clear in affirming the indications OS evaluated. Regarding accuracy assessment of the surgical guide, none of the studies determined whether there was any distortion in the surgical guide, and none even used the appropriate statistical test (ROC curves), which allows defining the true accuracy.

Regarding the reference standard, all studies showed a high risk of bias because they compared preoperative planning with the results obtained. However, we believe that accuracy cannot be analyzed in this approach. Most of the selected studies (n=11) compared the pre- and postoperative values, obtaining low risk of bias for different planning types. Only one study did not present this comparison. Regarding the time and flow of the surgery, all studies demonstrated low risk of bias, since there were appropriate intervals from the virtual planning to the surgery outcomes (15).

Considering that possible sources of error may be related to the accuracy of the final result of the post-osteotomy treatment, in addition to the guide, mention should be made of the internal and external reference points (20). Internal reference points are arbitrary anatomical points inserted by the surgeon in the maxilla bone, cranial and caudal, to plan the lines that will be performed for the osteotomy, as well as in the lateral walls of the maxilla during intraoperative planning. On the other hand, the external reference points measure the distance between the incisal edge of the incisor and a screw inserted in the nasion point, facilitating a more accurate positioning of the anterior maxilla (20).

Furthermore, the order of movement of the jaws has been suggested as an interference in the final accuracy of OS. Thus, when evaluating the performance of Le Fort I osteotomy, it is currently possible to choose the initial positioning, as a guide to the maxilla or mandible. Although most surgeons elect the maxilla to the first movement, because it was the first technique applied, the mandible is also being repositioned through a more advantageous technique when it is necessary to compensate for the error in condylar positioning. Despite this, there is a consensus in the literature that the maxilla is still the most adequate for initial movement (20-23).

## Conclusions

The selected studies provided insufficient and heterogeneous information. Moreover, can be observed that the analysis of the accuracy of virtual guides for OS was not performed with the statistical test of the ROC curves, and therefore, the true accuracy of the guide was not determined. The planning and printing errors related to the guide were all less than 2 mm and the absolute averages of the errors related to virtual planning in the analysis of the different planes, namely sagittal, vertical, horizontal, axial, vertically, anteroposteriorly, and mediolaterally, were less than 1 mm. Finally, the measurement of the ANB angle achieved equivalent results between virtual and traditional planning.
